# Changes in Dietary Patterns from Childhood to Adolescence and Associated Body Adiposity Status

**DOI:** 10.3390/nu9101098

**Published:** 2017-10-06

**Authors:** Danielle Biazzi Leal, Maria Alice Altenburg de Assis, Patrícia de Fragas Hinnig, Jeovani Schmitt, Adriana Soares Lobo, France Bellisle, Patrícia Faria Di Pietro, Francilene Kunradi Vieira, Pedro Henrique de Moura Araujo, Dalton Francisco de Andrade

**Affiliations:** 1 Post Graduate Program in Nutrition, Health Sciences Center, Federal University of Santa Catarina, CCS/UFSC, Campus Trindade, Florianopolis 88040-900, Brazil; malicedeassis@gmail.com (M.A.A.d.A.); phinnig@yahoo.com.br (P.d.F.H.); adri_lobo@hotmail.com (A.S.L.); fariadipietro@gmail.com (P.F.D.P.); frankunradi@gmail.com (F.K.V.); 2 Post Graduate Program in Physical Education, Sports Center, Federal University of Santa Catarina, CDS/UFSC, Campus Trindade, Florianopolis 88040-900, Brazil; 3 Post Graduate Program in Production Engineering, Technological Center, Federal University of Santa Catarina, University Campus Trindade, Florianopolis 88040-900, Brazil; jeovani.schmitt@yahoo.com.br (J.S.); phma05@gmail.com (P.H.d.M.A.); 4 Equipe de Recherche en Epidémiologie Nutritionnelle, Centre de Recherche en Epidémiologie et Statistiques, Université Paris 13, Inserm (U1153), Inra (U1125), Cnam, COMUE Sorbonne Paris Cité, Bobigny 93017, France; bellisle@uren.smbh.univ-paris13.fr; 5 Informatics and Statistics Department, Technological Center, Federal University of Santa Catarina, University Campus Trindade, Florianopolis 88040-900, Brazil; daltoncasa@hotmail.com

**Keywords:** dietary patterns, tracking, children, adolescents, factor analysis

## Abstract

The aims of this study were to identify cross-sectional dietary patterns (DPs) in a representative sample of 7–10-year-old schoolchildren, to examine how scores for these DPs tracked over a time period of five years (from age 7–10 years to 12–15 years), and to investigate longitudinal associations between changes in DPs scores and changes in BMI (Body Mass Index) *z*-scores. Children aged 7–10-years were examined in 2007 (*n* = 1158) and a subset of the sample participated in a follow-up in 2012 (*n* = 458). Factor analysis (FA) was applied to derive DPs at baseline. The change in DP from childhood to adolescence was analyzed by comparing factor scores using the complete cases, in which factor loadings were the ones evaluated at baseline. Associations of BMI change with DP change were assessed by multivariate linear regression. At baseline, four DP were identified that explained 47.9% of the food intake variance. On average, the factor scores of “DP II” (salty snacks, French fries, fast-food, sugary beverages) decreased in follow-up, while no changes were observed for “DP I” (rice, cooked beans, beef/poultry, leafy vegetables), “DP III” (fruits, cooked and leafy vegetables, fruit juices, pasta, milk, cheese), and “DP IV” (milk, coffee with milk, cheese, breads/biscuits). No significant linear association was shown between changes in BMI *z*-scores and changes in DP scores from childhood to adolescence. In conclusion, three out of four DP scores identified at baseline tracked slightly in adolescence.

## 1. Introduction

Establishing healthy eating habits is important during childhood and adolescence, given that these behaviors may have cumulative effects on health and tend to be continued into adulthood [1,2]. The transition in diet from childhood to adolescence marks important changes due to individual factors such as physiological development related to growth and maturation, changes in parental influence, as well as the increasing independence and interaction of adolescents with their social environment [3,4]. Investigating eating behaviors in children longitudinally during the transition into adolescence is important in order to provide information on the nature of individual-level change over time and when, how, and why dietary changes occur [4].

The transition in a child’s diet may be affected by socioeconomic and demographic changes within the family [4,5]. In addition, in many emerging and developing countries, as incomes rise and populations experience urbanization, there is a shift from traditional fiber and grain-rich diets to fat, sugar-rich, refined grains, animal fat, and protein diets [6], leading to obesity and diet-related chronic diseases [7].

In epidemiology, tracking is defined as the stability or maintenance of a given constant over time [8]. Dietary tracking values can therefore be considered to demonstrate the preservation of dietary habits, and the consumption of food or nutrients over a period of time [1]. Dietary patterns (DPs) are found to track during infancy [9], to older childhood [10], and from children to adults [1], and then remain stable in adulthood [11]. In children and adolescents there are mixed findings, with some studies reporting stability [1,4,12,13], and others showing changes in DPs [14,15,16,17]. The limited available prospective epidemiological evidence consistently indicates that DPs that are high in energy-dense, high-fat, and low-fiber foods predispose young people to overweight and obesity later in life [18,19,20].

DPs have been identified in some cross-sectional studies in Brazilian children and adolescents [21,22,23,24,25,26]. None of these studies has investigated the stability of DPs specifically from childhood to adolescence, and their relationship with changes in body mass index. 

We have previously reported the association between dietary patterns (derived by latent class analysis) and overweight/obesity in a cross-sectional study of 7 to 10-year-old Brazilian schoolchildren [25]. In the present study, we assessed the stability of DPs in the same children five years later, using confirmatory factor analysis (FA). FA was also used at baseline, as this is currently the most popular method for identifying DPs.

The aims of the present study were (a) to identify cross-sectional DPs in a representative sample of schoolchildren aged 7–10 years old; (b) to examine how scores for these DPs tracked over a time period of five years (from age 7–10 years to 12–15 years) and (c) to investigate longitudinal associations between changes in DPs scores and changes in body mass index (BMI) *z*-scores.

## 2. Materials and Methods

### 2.1. Design and Study Population

A cross-sectional survey designed to investigate the prevalence of overweight/obesity and related behaviors of schoolchildren was conducted in Florianopolis (Brazil) from April to October 2007. The final sample consisted of 1232 children from 17 schools (782 children from 11 public schools and 450 children from six private schools). Detailed sampling procedures have been described elsewhere [27].

In 2012, an active search of all adolescents (12–15 years) surveyed in 2007 was performed using the Brazilian School Census (EducaCenso). However, the collection of data for this second study was restricted to students still enrolled in the same schools as the 2007 survey or transferred to other schools in the metropolitan area of the city. As the schoolchildren search did not occur in other regions of the state or the country, the eligible sample decreased. A total of 494 cohort members from 65 schools were identified and interviewed in 2012 (40.1% of the 2007 participants). 

**Figure 1:**
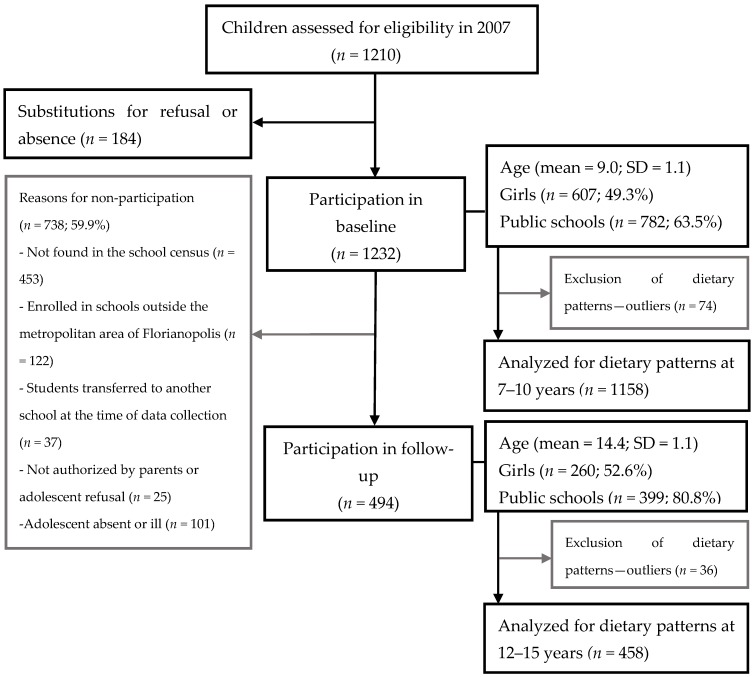
Flowchart of participants in the 2007 survey (7–10-year-olds) and in the 2012 survey (12–15-year-olds).

After excluding 74 children at baseline (boys *n* = 44, girls *n* = 30) and 36 adolescents at follow-up (boys *n* = 21, girls *n* = 15) with outlier’s data for food intake (i.e., reporting fewer than three food items or with a total daily frequency intake of foods/beverages exceeding three standard deviation scores), 1158 children (581 boys and 577 girls) at baseline and 458 adolescents (213 boys and 245 girls) at follow-up were included in the dietary pattern analyses. Detailed sampling and reasons for follow-up losses are shown in the flowchart (Figure 1).

Both studies were conducted according to the guidelines set out in the Code of Ethics of the World Medical Association (Declaration of Helsinki) and all procedures involving human subjects were approved by the Human Studies Committee of the Federal University of Santa Catarina (07636813.3.0000.0121). Written informed consent was obtained from the parents and oral assent was obtained from the children.

### 2.2. Dietary Intake

At both time points, dietary data were obtained using the third version of the Previous Day Food Questionnaire (PDFQ-3) [28] based on a single day recall procedure, designed to investigate the consumption frequency of specific foods (not nutrients) as markers of (un)healthy diet and types of physical activities on the previous day. For example, a range of specific foods have been suggested as important dietary determinants of weight status in childhood and adolescence, including fruit and vegetables [29], fat [30], fast-food [31], and sugary drinks [32]. The PDFQ is a paper and pencil questionnaire, designed to be applied in the school setting as a supervised classroom exercise where children are guided by trained researchers following a standardized protocol [28]. The PDFQ-3 was previously validated in a sample of 6–11-year-old schoolchildren, with direct observation of the food eaten at school meals on the previous day as the gold standard, and demonstrated a reasonable average sensitivity (probability of correctly reporting a food intake) of 70.2% and an excellent average specificity (probability of correctly not reporting a food intake) of 96.2% [28]. The food section of the questionnaire covers six daily eating occasions (three main meals and three snacks) ordered chronologically (breakfast and mid-morning snack, lunch and afternoon snack, dinner and evening snack). Each meal and snack is illustrated with 21 pictures of foods/beverages or food groups (bread and biscuits, chocolate milk, coffee with milk, milk, yoghurt, cheese, rice, beans, pasta, beef and poultry, fish and seafood, leafy vegetables, cooked vegetables, vegetable soup, fruits, fruit juices, French fries, pizza and hamburgers, sweets, salty snacks, and soft drinks). The tool does not assess foods or food groups like water, cooking fats, fat, or sugary spreads on bread (e.g., butter or margarine, honey, jam, chocolate or nut-based products), fat content (e.g., low-fat milk or high-fat milk), types of soft drinks and fruit juices (e.g., regular or diet), or types of cooking methods (e.g., frying, baking, roasting).

The foods and food groups illustrated in PDFQ-3 were selected in order to represent the food patterns of children in this age group, foods presented in school menus, and foods recommended in the guidelines for the Brazilian population [33]. Supplementary Figure S1 shows one page of the questionnaire.

The PDFQ-3 was assessed once at each time point for every child, and the day at which the PDFQ-3 was assessed differed between children. This strategy was used in order to describe the daily variability of dietary intake on schooldays (Monday to Thursday) and non-school days (Sunday and holidays) allowing for the analysis of food consumption at the group level. In the 2007 survey, 71.3% (*n* = 826) of the participants reported food intake on weekdays/schooldays (16.8% on Monday; 22.1% on Tuesday; 17.2% on Wednesday; 15.2% on Thursday), and 28.7% (*n* = 332) on Sunday/holidays (non-schooldays). As the PDFQ-3 was applied in the school setting and there was no school on Saturdays and Sundays, it was not possible to obtain data representing food consumption for Fridays and Saturdays. Portion sizes were not assessed; therefore, the food intake could not be quantified by weight or energy and instead the (relative) frequency of intake was used as an indicator for the factors. However, number of servings per day (frequency) is routinely used to determine empirical dietary patterns [34,35,36]. The frequency of food intake was estimated as number of times per day, ranging from 0 to 6 for each food/beverage consumed, assuming that only one serving was consumed on each occasion. 

### 2.3. Physical Activity

In the physical activity section of the PDFQ-3, schoolchildren were asked to report their physical activities (walking/running, playing with a dog, cycling, swimming, playing ball games, jumping rope, athletics, climbing stairs, roller skating/blading, dancing, and helping with household chores). The validation of the physical activity section of the questionnaire using comparisons between the scores generated by the instrument and the number of step counts obtained by pedometers showed mean values for sensitivity and specificity of 78% and 56%, respectively [37]. In the present study, metabolic equivalents (METs) were assigned to each activity reported using the Compendium of Energy Expenditures for Youth [38] and summed for all physical activities (PA) reported by each child. PA in terms of metabolic equivalents (PA MET) were categorized into tertiles (the first tertile was defined as lowest, second tertile as intermediary, and third tertile as highest METs). This scoring method was validated in Brazilian children in a previous study [39].

### 2.4. Anthropometric and Sociodemographic Measurements

The administrative department of each school provided information on the child’s date of birth, sex, and type of school (an important marker of socioeconomic condition in Brazil). Trained research staff measured weight and height of participants following standard techniques [40] in both surveys. Theoretical and practical workshops on measurement techniques were held to standardize the anthropometric measurements in both surveys [41]. Anthropometric measurements were taken with the children wearing light clothes and without shoes. Weight was measured with a digital 180 kg scale (Marte^®^, model PP, 50 g precision). Height was measured with a portable stadiometer (Alturexata^®^, 1 mm precision). Body mass index (BMI) was computed as weight (in kg) divided by the square of height (in m). Parents completed a self-administered questionnaire reporting their weight, height, and monthly family income. Monthly family income was defined as a categorical variable taking into account the minimum wage at both time points (<3; ≥3 and <5; ≥5 and <10; ≥10). Maternal weight status was assessed by BMI based on self-reported weight (kg) and height (m). Type of school was constructed as a dichotomous variable (public or private). Children’s age was computed as the difference between the date of birth and the date of measurements. 

At both time points, children’s BMI data was converted into *z*-scores (according to age and sex) based on the World Health Organization Growth References (WHO-2007) [42]. Weight status of children (baseline) and adolescents (follow-up) were then categorized as non-overweight (N-OW) (BMI-for-age < +1.0 SD) or overweight including obesity (OW) (BMI-for-age ≥ +1.0 SD). Underweight (0.8%) and normal weight children were grouped together into the N-OW category, while overweight (non-obese) and obese (6.7%) children were grouped into the OW category. Weight status change was based on individual changes from childhood to early adolescence. In the regression analysis, the change in BMI *z*-score (BMI *z*-score at follow-up − BMI *z*-score at baseline) was modeled as a continuous variable in order to consider the entire distribution of weight status among the whole study population.

### 2.5. Statistical Analysis

In order to identify DPs in a representative sample of 7–10-year-old schoolchildren, exploratory FA with principal component estimation was applied to the total sample at baseline (*n* = 1158). A polychoric correlation model for categorical ordered data was used. The number of factors to retain was first examined by the chi-squared test, which showed that the five-factor solution provided the best fit of the a priori model (baseline FA) (χ^2^(12) = 31.51, *p* < 0.01). However, the four-factor solution produced better interpretability, which was confirmed by the common practice to choose components with Eigenvalues >1.5 to limit the factors [43], and by the examination of the Scree plot (Figure S2). After the choice of the number of factors, the factor loadings of foods items were calculated. Those foods/beverages that showed low loadings for all factors were excluded from the analysis (yoghurt, sweets, fish and vegetable soup), as they did not explain any factor. After the exclusion of those foods, FA was performed again, and four major dietary patterns were considered as best representing the data. The varimax orthogonal rotation was carried out in order to simplify the interpretation of the data, maximizing the higher factor loadings and minimizing the lower ones. Variables with factor loadings ≥0.30 or ≤−0.30 were considered important for the interpretability of the factors. For the total sample in 2007, the factor scores for each DP were calculated at the individual level by summing the observed standardized frequencies of consumption per food/beverage, weighted according to the absolute factor loadings. The factor scores were standardized and the group mean factor scores were set to zero.

A confirmatory analysis for the complete cases (*n* = 458) in 2012 was used, in which all factor loadings were the ones evaluated at baseline. This allowed the comparisons in mean factor scores between the same children at follow-up. Using this approach, the changes in factor scores reflected actual differences in the intake frequency of foods/beverages identifying the factor (pattern) rather than a change in the individuals’ relative rank position compared to the group mean intake frequency [44]. A high factor score for a given pattern indicated high frequency intake of the foods constituting that food pattern, and a low score indicated low frequency intake of those foods. Factors were labeled by numbers (I, II, III, IV) according to the variance explained and were interpreted according to the food groups that loaded highly on each pattern.

Tracking was defined as how stable the factor scores identified at baseline in the complete-cases sample remained at follow-up. Paired *t* test was used to determine whether the mean factor scores difference between the completers was zero. Spearman’s correlation was calculated between the factor scores obtained at each time point. The power analysis of these effects was analyzed considering the effect size measured with Cohen’s *d* parameters: <0.2 = small effect, 0.2 to 0.8 = medium, and >0.8 = large [45].

Multivariate linear regression was used to examine the changes in BMI *z*-scores (defined as the difference between BMI *z*-score at follow-up and at baseline) associated to changes in each dietary pattern scores (the difference between dietary patterns scores at follow-up and at baseline), simultaneously. The potential confounding variables in the multiple regression models were: child’s age (continuous), sex, BMI *z*-scores (continuous), maternal BMI (continuous), type of school (private or public), monthly family income (categorical), day of the week (school days or Sunday/holidays), and tertiles of PA MET at baseline. The maternal BMI was included in the model, as there is a solid body of evidence on both genetic and environmental parental influence on children’s dietary patterns [9,23]. All the covariates were chosen among a range of possible confounders because they were both associated with BMI and with food intake. Interaction terms were not used, as preliminary analyses did not find important examples of interaction, as well as to avoid over-adjustment and chance findings (data not shown).

To investigate if the DPs differed on school days compared with non-school days (Sunday and holidays), exploratory FA with principal component estimation was applied to baseline data for children who reported their food intake on school days using the same analytical approach described above for the complete baseline data. The factor scores obtained on school days were standardized and the group mean factor scores were set to zero and used as a reference. Then, the factor scores for non-school days were computed in a simple confirmatory FA model, in which the loadings on the four factors were those evaluated at school days. One-sample *t* test was used to test the differences between the mean factor scores for non-school days versus school days. 

Considering that the sample size available for analyses was restricted to complete cases (*n* = 458), with 80% test power and alpha error of 5%, the study was able to detect an effect size of at least 0.131 for the two-tail paired *t*-test; at least 0.165 for the one-tail Spearman’s rho; and at least 0.027 in the multivariate linear regression. 

Statistical significance level was set at 5%. Statistical software R [46] was used for factor analysis and Stata 13.0 (Stata Corp., College Station, TX, USA) was used for descriptive and analytical statistics.

## 3. Results

The total baseline characteristics of participants at age 7 to 10 years (*n* = 1158), including children participating in the follow-up survey (*n* = 458) or lost to follow-up (*n* = 700), are shown in Table 1. There were no differences for age, sex, BMI, prevalence of overweight, mother’s weight status, monthly family income, day of the week, and tertiles of PA MET. Nevertheless, a greater proportion of children enrolled in private schools were lost in the follow-up.

After computing the FA on the 17 foods/beverages at baseline, four DPs were identified with the highest Eigenvalues that accounted for 47.9% of the total variance of food intake and could be interpreted meaningfully in terms of nutritional characteristics. The detailed structures of the four DPs with their explained variance and loading coefficients are shown in Table 2. The “DP I” had positive high loadings on rice, cooked beans, leafy vegetables, and beef/poultry, while having negative loadings on pasta and fast-food, suggesting that the intake of these foods showed a deviation from this DP. The high loading foods on “DP II” included French fries, salty snacks, soft drinks, and fast-food. The “DP III” loaded highly on fruit juices, cooked vegetables, fruits, pasta, leafy vegetables, cheese and milk, with a negative loading on soft drinks. Finally, the “DP IV” loaded highly on coffee with milk, breads/biscuits, cheese and milk, with negative loadings on chocolate milk and fast-food (Table 2).

Table 3 shows mean factor scores of the four dietary patterns at the two time points in the complete cases (*n* = 458), spearman correlations coefficients between factor scores at the two time points, and the effect size for each analysis. Considering the difference between the mean factors scores at the two time points, our sample allows us to conclude that only the scores for DP II differed between the two time points, with a medium effect size. The sample size of the follow-up has sufficient power to show correlations between factor scores of DPs I, III, and IV at the two time points, with a medium effect size. These findings together imply that children presenting higher scores for DP I, DP III, and DP IV at baseline also showed higher scores for the respective DPs at follow-up, and the mean frequencies of consumption of foods constituting these patterns did not increase. On the other hand, no correlation was found for DP II scores between baseline and follow-up and the mean frequency of consumption of the foods constituting the pattern decreased (Table 3).

After controlling for baseline BMI *z*-scores, maternal BMI, sex, age, type of school, family income, day of the week, and tertiles of PA MET, there was no significant linear association between changes in BMI *z*-scores and changes in DP scores from childhood to adolescence. The analysis of the change in DP scores showed a small effect size on the change of BMI *z*-scores (Table 4). Based on the weight status categories defined by WHO-2007, out of the 153 children classified as overweight or obese at baseline, 65.4% (*n* = 100) remained overweight/obese in adolescence (boys: 69.8%; girls: 59.7%). Out of the 297 children classified as non-overweight at baseline, 9.4% (*n* = 28) were found to be overweight/obese (boys: 4.8%; girls: 9.8%) at follow-up.

The FA on the same 17 foods/beverages applied at baseline in the sample who reported food intake on school days also identified four DPs with higher Eigenvalues that explained 49.0% of the food intake variance (Table S1). One-sample *t* test showed that the two DP scores (DP I and DP II) for non-school days were statistically different from zero, i.e., food intake on non-school days differed from school days for these patterns (Table S2).

## 4. Discussion

The longitudinal analysis of the present study conducted with the complete cases of children and adolescents five years apart showed that, on average, the factor scores for “DP II” (French fries, salty snacks, soft drinks and fast-food) identified at baseline (in 7–10-year-old children) decreased in the follow-up sample (in 12–15-year-olds), whereas no changes were observed for “DP I” (rice, cooked beans, leafy vegetables, beef/poultry), “DP III” (fruit juices, cooked vegetables, fruits, pasta, leafy vegetables, cheese and milk), and “DP IV” (coffee with milk, breads/biscuits, cheese and milk).

Cross-sectional analysis of data obtained at baseline identified four DPs that satisfactorily captured eating behavior (47.9% of food intake variance explained) in this population-based sample of Brazilian schoolchildren. These DPs were also identified in our previous study that extracted DPs by Latent class analysis (LCA) based on the time-of-day of eating events [25] and resembled other patterns identified in Brazilian and international studies conducted in children and adolescents. “DP I” in the present study (including rice and beans) was also identified in studies based on the Brazilian Household Budget Surveys (2002–2003) [47] and (2008–2009) [23]. The latter, conducted in individuals over 10 years of age, confirmed the aggregation of DPs among members of the same family and extracted three major DPs by factor analysis: “Traditional snack” (coffee, rolls, oils and fats, cheese), “Traditional main meal” (rice, beans and other legumes, and meat) and “Fast-food snack” (sandwiches, processed meats, soft drinks, snacks, pizza) [23] in line with “DP I” and “DP II” (salty snacks, French fries, fast-food and sugary beverages) of the present study. The “DP II” in the present study shared various dietary items of high fat and high energy-density items with other studies of children and adolescents in Brazil [21,22], Colombia [48], Canada [49], and European countries [1,35,50,51]. Our “DP III” (fruit juices, cooked vegetables, fruits, pasta, leafy vegetables, cheese and milk) was similar to the “health aware/conscious” pattern associated with lower fat gain in girls between 9 and 11 years of age in the Avon Study in England [52,53].

On average, the factor scores for “DP II” (French fries, salty snacks, soft drinks, and fast-food) identified at baseline (in 7–10-year-old children) decreased in the follow-up sample (12–15-year-olds), whereas no changes were observed for “DP I” (rice, cooked beans, leafy vegetables, and beef/poultry), “DP III” (fruit juices, cooked vegetables, fruits, pasta, leafy vegetables, cheese and milk), and “DP IV” (coffee with milk, breads/biscuits, cheese and milk). Although food consumption was only assessed for one day in the present study and considerable within-subject variation in daily food consumption may occur, our results provide evidence of slight stability of three DP scores (DP I, III, IV) as well as change in “DP II” scores over the five-year follow-up period. 

Direct comparisons of the present results with previous DP studies in children and adolescents should consider differences in study design, sample sizes, dietary assessment methods, and statistical methods to derive DPs and to estimate the tracking of DPs. Furthermore, the extent of changes of DP from childhood to adolescence may vary in different populations due to cultural, social, and economic factors. A review of the literature found that the tracking of DPs ranged from weak to moderate between childhood and adolescence [54].

The stability of the mean factor scores for “DP I” (rice, cooked beans, beef/poultry, leafy vegetables) and “DP IV” (milk, coffee with milk, cheese, and breads/biscuits) from childhood to adolescence may be explained by cultural factors. The components of these patterns are traditional foods eaten in Brazilian meals (“DP I” in lunch and/or dinner; “DP IV” in breakfast and/or snacks between meals). These patterns were also identified in cross-sectional studies conducted with children [22,24,25], adolescents [21,22,26], and adults [55] from different regions in Brazil.

We expected to observe an increase in scores for “DP II” (salty snacks, French fries, fast-food, and sugary beverages) at follow-up. However, this was not the case in our study, and the decrease in its factor scores at follow-up might result from a social desirability bias (underreporting). It is also possible that adolescents at follow-up consumed larger portions of the unhealthy foods constituting “DP II”, but less frequently. We cannot investigate this hypothesis because the PDFQ-3 did not measure portion sizes. Therefore, energy intake could not be estimated and input variables for DP analysis could not be adjusted for energy intake. The issue of adjusting for energy intake is still controversial in studies of changes in DPs [34].

The present study did not find a significant linear association between changes in DP scores and changes in BMI *z*-scores. The longitudinal association of a high adherence to unhealthy DPs with increased risk of overweight and obesity was not verified in some earlier studies [12,56]. In the European DONALD study [57], a positive though small association between baseline consumption of high-energy convenience foods and the change in the percentage of body fat over a five-year follow-up was found among boys. A cohort of 5–12-year-old children from low- and middle-income families in Bogota showed that those in the highest quartile of adherence to a snacking pattern had a 0.09 kg/m^2^ higher BMI gain and 0.012 mm higher gain in trunk adiposity per year compared to children in the lowest quartile [48]. 

The strengths of the present study were the longitudinal follow-up design based on a relatively large sample size at baseline, the use of the same validated questionnaire to assess food intake at the two time points, the use of potential confounding variables in the regression analysis, and the application of confirmatory FA to compute factor scores over time. An important feature of our analysis, which was rarely present in previous longitudinal studies on the tracking of DPs [19,44], is that we applied confirmatory FA to calculate factor scores at follow-up by using the factor loadings obtained at baseline. By using this approach to compute the factor scores, the problem of the data dependency and lower reproducibility of factors in different datasets is eliminated [44]. When exploratory FA is used to identify DPs and to investigate their stability over time, the correlation between foods is commonly used to create multiple continuous scores at each time point. A difficulty inherent in this approach is that the DPs obtained are not generally reproducible. The performance of separate exploratory FA at each time point to evaluate tracking may result in high correlations between pattern scores at baseline and at follow-up, despite large changes in the correlations between the specific food items that define a pattern [11].

We acknowledge some limitations in the present study. First, the retention rate in the follow-up study (40.1%) was low, limiting the generalizability of our results to the general population. It could be argued that changes in DP scores from childhood to adolescence were a result of attrition biases. However, children lost to follow-up did not differ significantly from the included participants in DP scores, demographic variables, and weight status at baseline. Therefore, we believe that the low correlations found between the baseline factor scores of DPs I, III, and IV and its scores at follow-up were not affected by such bias. We cannot exclude the possibility that the lack of association found between changes in DPs and changes in BMI *z*-score could be due to the underrepresentation of adolescents who studied in private schools (wealthier than adolescents from public schools in Brazil) in the follow-up study.

Second, as for all other dietary assessment studies, the self-reported food recall may potentially be subject to misreporting. The PDFQ-3 used in this study proved to be a valid instrument [28] and has been used to assess dietary patterns and behaviors [25,26,58]. The PDFQ-3 was designed to avoid the difficulties associated with children’s assessments of portion size, and to simplify the memory task by prompting only the relevant food items eaten on the previous day. The cognitive task required for estimating portion size, frequency, and averaging may not be compatible with the perceptual and conceptual capacities of children who have not reached the stage of abstract reasoning at approximately 10–11 years of age [59,60]. Like some questionnaires validated in other countries [35,61], this approach keeps the questionnaire relatively brief and easy for the child to complete with minimal assistance. Also, the dietary information is derived from the children themselves (without help from parents or guardians). The use of the parent report of a child’s diet has also been seen as a limitation [35,62]. In the age range of the present study (7–15 years), children spend a considerable amount of time unsupervised by their parents, who therefore could not validate the children’s dietary recall.

The results are based on the frequency of food/beverage and food groups eaten on only one day in each time point in a group of children and conclusions may be applicable to the population rather than to individuals, since a single day of intake may not be representative of usual individual intake. The expected effect of use a food questionnaire that only covers one day of dietary intake is to attenuate the statistical association between changes in adherence to a dietary pattern and changes in BMI *z*-score [15,63].

The differences in DP between weekdays/schooldays and non-schooldays found in the present study should be highlighted, and in this sense our results are strengthened by a good coverage of the days of the week (school days), including one day of the weekend (Sunday) and holidays to cover the variation in diet at the group level. While the factor scores for “DP I” on weekdays/school days with high loadings for unhealthy foods (fast-foods, French fries, salty snacks, and soft drinks) increased on Sunday/non-school days (*p* < 0.01), the factor scores for “DP II” with high loadings for foods included in Brazilian traditional lunch and/or dinner (rice, beans, beef/poultry) decreased on Sunday/non-school days (*p* < 0.01), thus indicating that specific foods eaten on non-schooldays were less healthy. Changes in daily patterns such as not attending school on the weekend contribute significantly to changes in dietary patterns of food consumption, patterns of physical activity, and ultimately energy balance [64]. Previous research in children indicates that dietary quality is poorer on the weekends compared with weekdays, with significantly higher intakes of total sugars [62], sugar sweetened beverages, confectionery, and lower consumption of fruit and vegetables [62,65]. The potential influence of day-to-day variation of food consumption in the FA merits more attention in future research.

In future studies, information on children’s diet from the PDFQ-3 should be integrated with a food frequency questionnaire filled in by parents to complement the measure of food items not covered by the child’s questionnaire. In addition, the PDFQ-3 should be applied on various days of the week, including one day of the weekend at each time point, assuring that the child completes the PDFQ-3 on the same day of the week at each time point.

Additionally, although we adjusted for a variety of potential confounding variables, residual confounding cannot be ruled out. In particular, adjustments for social mobility, which expresses life-long changes in family income [5], and which might affect dietary habits and changes in weight status, were not possible due to the lack of information.

## 5. Conclusions

Four dietary patterns were identified at baseline in schoolchildren aged 7–10 years old: “DP I” (rice, cooked beans, beef/poultry, leafy vegetables), “DP II” (salty snacks, French fries, fast-food, sugary beverages), “DP III” (fruits, cooked and leafy vegetables, fruit juices, pasta, milk, cheese) and “DP IV” (milk, coffee with milk, cheese, breads/biscuits). Slight tracking was observed between factor scores for “DP I”, “DP III”, and “DP IV” from childhood to adolescence using the complete cases, while the factor scores for “DP II” decreased in follow-up. No significant linear association was shown between changes in BMI *z*-scores and changes in DP scores over a five-year period.

We cannot be certain that the level of tracking of dietary patterns found in the present study reflected a real finding or resulted from an artifact due to change in intake from a single day in childhood to a single day in adolescence. We intend to address this in the next follow-up of the study by taking repeated dietary assessments during childhood and adolescence.

## Figures and Tables

**Table 1:** Characteristics of the study participants by follow-up status.

	Not Followed-Up (*n* = 700)	Followed-Up (*n* = 458)	Total Baseline (*n* = 1158)
	Mean ± SD (95% CI)		
**Age (years)**	9.1 ± 1.2 (8.9–9.1)	9.0 ± 1.1 (8.9–9.2)	9.0 ± 1.1 (8.9–9.1)
**BMI**	17.8 ± 2.8 (17.5–18.0)	17.6 ± 2.9 (17.2–17.8)	17.7 ± 2.9 (17.5–17.7)
	**% (95% CI)**		
**Sex**			
Boys	49.4 (45.7–53.1)	46.5 (42.0–51.1)	48.3 (45.4–51.2)
Girls	50.6 (46.9–54.3)	53.5 (48.9–58.0)	51.7 (48.8–54.6)
**Overweight ^a^**			
Yes	34.3 (30.9–37.9)	34.3 (30.1–38.8)	34.3 (31.6–37.1)
No	65.7 (62.1–69.1)	65.7 (61.2–69.9)	65.7 (62.9–68.4)
**Type of school**			
Public	70.4 (66.9–73.7)	86.2 (82.8–89.1)	76.7 (74.2–79.0)
Private	29.6 (26.3–33.1)	13.3 (10.9–17.2)	23.3 (21.0–25.8)
**Mother’s weight status ^b^**			
Thin	3.5 (2.3–5.2)	5.5 (3.7–8.1)	4.3 (3.2–5.7)
Normal weight	65.9 (62.2–69.4)	63.0 (58.4–67.5)	64.8 (61.9–67.6)
Overweight	22.6 (19.5–25.9)	20.8 (17.2–24.9)	21.9 (19.5–24.4)
Obese	8.0 (6.2–10.4)	10.6 (8.0–13.9)	9.1 (7.5–10.9)
**Monthly family income (minimum wage) ^c^**			
<3	45.4 (41.4–49.5)	47.3 (42.4–52.3)	46.2 (43.1–49.3)
3–5	20.0 (16.9–23.4)	26.8 (22.7–31.4)	22.7 (20.2–25.5)
5–10	19.9 (16.9–23.4)	15.2 (12.0–19.1)	18.0 (15.8–20.6)
>10	14.7 (12.0–17.8)	10.7 (7.9–14.1)	13.1 (11.1–15.3)
**Day of the week ^d^**			
Non-school days	32.9 (29.5–36.4)	22.3 (18.7–26.3)	28.7 (26.1–31.4)
School days	67.1 (63.6–70.5)	77.7 (73.7–81.3)	71.3 (68.7–73.9)
**Tertiles of PA MET**			
Lowest	35.0 (31.5–38.6)	32.5 (28.4–37.0)	34.0 (31.3–36.8)
Medium	32.4 (29.1–36.0)	33.0 (28.8–37.4)	32.6 (30.0–35.4)
Highest	32.6 (29.2–36.1)	34.5 (30.3–39.0)	33.3 (30.7–36.1)

PA MET: Physical activities in terms of metabolic equivalents; BMI: Body mass index; WHO: World Health Organization; ^a^ Overweight (including obesity BMI ≥ +1.0 *z*-scores—WHO-2007); ^b^ BMI based on self-reported data on weight and height and classified according to WHO recommendations (Thin—BMI <18.5 kg/m^2^, Normal weight—BMI 18.5–24.9 kg/m^2^, Overweight non-obese BMI 25–29.9 kg/m^2^, Obese—BMI ≥30 kg/m^2^); ^c^ 1 minimum wage = $US 204.30 ($BR 380): September 2007 exchange rate; ^d^ Day of the week. Missing mother’s weight status data (4.0%); Missing income data (15.3%); Missing child’s BMI (1.7%).

**Table 2:** Structures of four dietary patterns identified by factor analysis with principal component method in a representative sample of 7–10-year-old schoolchildren in 2007.

	Dietary Patterns 2007 (*n* = 1158)			
	DP I	DP II	DP III	DP IV
**Variance explained (%)**	17.5	10.7	10.0	9.7
Foods and food groups	**Factor loadings ^a^**			
Beans (cooked)	**0.66**	−0.22	0.16	0.08
Beef/poultry	**0.30**	0.08	0.08	0.07
Bread/biscuits	0.04	−0.07	0.19	**0.54**
Cheese	**−0.34**	−0.28	**0.40**	**0.41**
Chocolate milk	−0.17	−0.09	0.21	**−0.58**
Coffee with milk	0.01	0.05	−0.08	**0.90**
Fast-food	**−0.41**	**0.31**	0.04	**−0.31**
French fries	−0.07	**0.85**	0.04	−0.10
Fruit juices	0.07	−0.07	**0.71**	−0.07
Fruits	0.16	0.03	**0.50**	0.00
Leafy vegetables	**0.57**	0.11	**0.40**	0.00
Milk	0.08	−0.08	**0.34**	**0.36**
Pasta	**−0.46**	0.22	**0.49**	0.15
Rice	**0.84**	−0.09	0.08	0.04
Salty snacks	0.08	**0.84**	−0.05	0.09
Soft drinks	−0.27	**0.49**	**−0.32**	−0.02
Vegetables (cooked)	0.21	−0.15	**0.54**	0.04

^a^ Factor loading values in bold: ≥0.30 or ≤−0.30.

**Table 3:** Mean (standard deviation), spearman correlation coefficients, and effect size for corresponding factor scores at each time point in the complete cases (*n* = 458).

	DP I	DP II	DP III	DP IV
	**Mean (SD)**			
Baseline	−0.04 (1.2)	−0.02 (1.1)	0.07 (1.1)	0.03 (1.3)
Follow-up	0.06 (1.2)	−0.39 (1.0)	−0.07 (1.1)	−0.08 (1.1)
*p*	0.13	<0.01	0.03	0.15
Effect size	0.07	0.25	0.10	0.07
	**Spearman Correlation**			
Baseline vs. Follow-up	0.20	0.07	0.22	0.10
*p*	<0.01	0.12	<0.01	0.03
Effect size	0.41	0.14	0.45	0.20

**Table 4:** Relation between changes in BMI *z*-scores and changes in factor scores from ages 7–10 years to 12–15 years in the complete cases.

Change in Dietary Pattern	Change in BMI *z*-Scores ^a^		
	Coefficient	*p*	Effect Size
DP I	0.01	0.92	0.00
DP II	0.02	0.45	0.00
DP III	−0.04	0.19	0.00
DP IV	0.05	0.07	0.01

^a^ Outcome change in BMI *z*-scores (*n* = 450) over the five years since baseline; Adjusted by age (continuous), sex, BMI *z*-score, maternal BMI, type of school, family income, day of the week (school days or non-school days), and tertiles of PA MET (all baseline).

## Supplementary Materials

The following are available online at http://www.mdpi.com/2072-6643/9/10/1098/s1, Figure S1: The Previous Day Food Questionnaire (PDFQ-3), page 2., Figure S2: Scree plot for identification of dietary patterns (components) by factor analysis (FA) with principal component estimation, Table S1: Structures of four dietary patterns identified by factor analysis with the principal component method on baseline cross-sectional data on school days., Table S2: Factor scores on non-school days versus school days (reference) and significance of difference, as assessed by one-sample *t* test.
