# Supplementary material for: Changes in Dietary Patterns from Childhood to Adolescence and Associated Body Adiposity Status
Source: Nutrients. 2017 Oct 6;9(10):1098. doi: 10.3390/nu9101098 (PMC5691714; doi:10.3390/nu9101098)
Supplement: Supplementary file 1 [file nutrients-09-01098-s001.docx]

Supplementary Materials


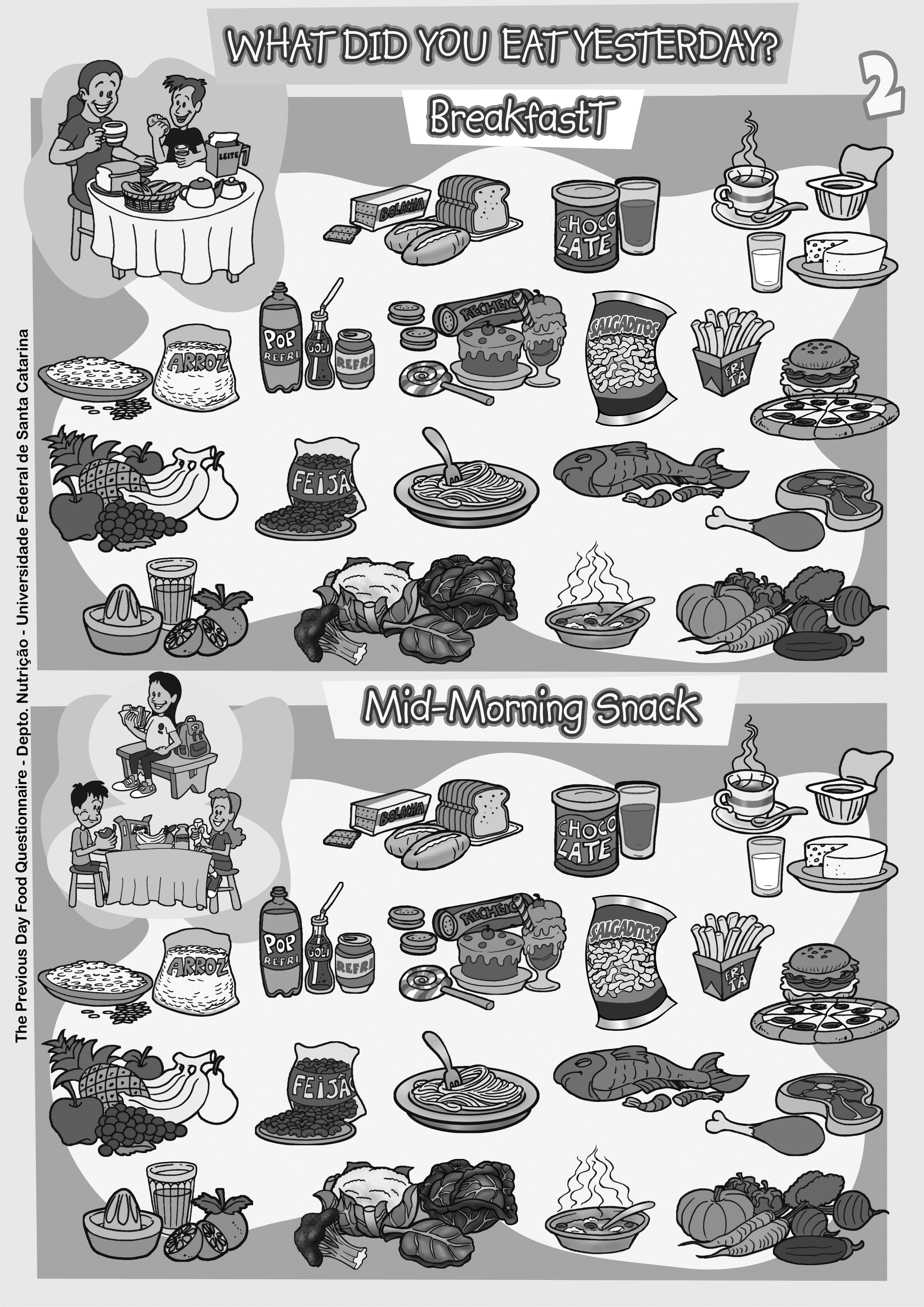


**Figure S1.** The Previous Day Food Questionnaire (PDFQ-3), page 2.

**Figure S2.** Scree plot for identification of dietary patterns (components) by factor analysis (FA) with principal component estimation.

**Table S1.** Structures of four dietary patterns identified by factor analysis with the principal component method on baseline cross-sectional data on school days.

| **Variance explained (%)** | **Baseline School Days (*n* = 826)** | | | |
| --- | --- | --- | --- | --- |
|  | **DP I** | **DP II** | **DP III** | **DP IV** |
|  | **15.0** | **12.0** | **11.0** | **11.0** |
| **Food and food groups** | **Factor loadings ^a^** | | | |
| Beans | −0.08 | **0.78** | 0.28 | 0.00 |
| Beef/poultry | −0.05 | **0.61** | **−0.39** | 0.27 |
| Bread/biscuits | −0.09 | 0.05 | **0.66** | 0.05 |
| Cheese | 0.02 | −0.03 | **0.81** | 0.09 |
| Chocolate milk | 0.12 | −0.05 | 0.15 | **−0.79** |
| Coffee with milk | 0.28 | 0.10 | **0.33** | **0.80** |
| Cooked vegetables | **−0.51** | 0.15 | −0.05 | −0.17 |
| Fast-food | **0.62** | −0.22 | 0.03 | −0.18 |
| French fries | **0.63** | 0.07 | 0.17 | 0.05 |
| Fruit juices | **−0.43** | 0.14 | 0.17 | 0.13 |
| Fruits | **−0.45** | 0.06 | 0.22 | −0.03 |
| Leafy vegetables | **−0.43** | 0.07 | **0.30** | −0.11 |
| Milk | **−0.37** | −0.03 | 0.23 | **0.43** |
| Pasta | −0.04 | **−0.32** | 0.04 | 0.23 |
| Rice | −0.18 | **0.90** | 0.00 | 0.09 |
| Salty snacks | **0.45** | 0.12 | **−0.34** | −0.12 |
| Soft drinks | **0.65** | 0.28 | 0.15 | −0.28 |

^a^ Factor loading values in bold: ≥0.30 or ≤−0.30.

**Table S2.** Factor scores on non-school days versus school days (reference) and significance of difference, as assessed by one-sample *t* test.

|  | **DP I** | **DP II** | **DP III** | **DP IV** |
| --- | --- | --- | --- | --- |
|  | Mean (SD) | | | |
| School days | 0.00 (1.27) | 0.00 (1.13) | 0.00 (1.24) | 0.00 (1.17) |
| Non-school days | 0.33 (1.31) | −0.24 (1.11) | 0.02 (1.24) | 0.04 (1.22) |
| *p* | <0.01 | <0.01 | 0.78 | 0.58 |
